# Recruitment, Infiltration, and Cytotoxicity of HLA-Independent Killer Lymphocytes in Three-Dimensional Melanoma Models

**DOI:** 10.3390/cancers13102302

**Published:** 2021-05-11

**Authors:** Ilenia Iaia, Loretta Gammaitoni, Giulia Cattaneo, Lidia Giraudo, Chiara Donini, Erika Fiorino, Luca Primo, Fabrizio Carnevale-Schianca, Massimo Aglietta, Alberto Puliafito, Dario Sangiolo

**Affiliations:** 1 Candiolo Cancer Institute, FPO-IRCCS, 10060 Candiolo, Italy; ilenia.iaia@ircc.it (I.I.); loretta.gammaitoni@ircc.it (L.G.); giulia.cattaneo@ircc.it (G.C.); lidia.giraudodiego@ircc.it (L.G.); chiara.donini@ircc.it (C.D.); erika.fiorino@ircc.it (E.F.); luca.primo@ircc.it (L.P.); fabrizio.carnevale@ircc.it (F.C.-S.); massimo.aglietta@ircc.it (M.A.); alberto.puliafito@ircc.it (A.P.); 2 Department of Oncology, University of Turin, 10060 Candiolo, Italy

**Keywords:** 3D models, melanoma, cell therapy, imaging

## Abstract

**Simple Summary:**

Limited therapeutic results of immune checkpoint inhibitors in definite tumor settings, such as melanoma, call for alternative or complementary approaches. Among these, adoptive cell therapy (ACT) by means of HLA-independent tumor killer lymphocytes is a promising approach. We aimed at developing a pre-clinical 3D model to investigate and visualize the interaction between tumor and immune effectors in melanoma. To this aim, we employed Cytokine-Induced Killer cells (CIK) and NK-92 on patient-derived melanoma samples. By means of imaging-based methods, we measured the effector recruitment on the 3D targets, their infiltration, and cytotoxic activity. Our results and methodologies can be easily generalized to other effectors and other classes of tumors and help elucidate fundamental questions on the basic biology and kinetics of immune effector recruitment in a realistic 3D setting mimicking a solid tumor.

**Abstract:**

Cancer adoptive cell therapy (ACT) with HLA-independent tumor killer lymphocytes is a promising approach, with intrinsic features potentially addressing crucial tumor-escape mechanisms of checkpoint inhibitors. Cytokine-induced Killer (CIK) and Natural Killer (NK) lymphocytes share similar tumor-killing mechanisms, with preclinical evidence of intense activity against multiple solid tumors and currently testing in clinical studies. To improve the effective clinical translation of such ACT approaches, several fundamental questions still need to be addressed within appropriate preclinical contexts, capable of overcoming limitations imposed by most traditional two-dimensional assays. Here, we developed a novel experimental approach to explore, dissect, and visualize the interactions of CIK and NK lymphocytes with melanoma tumors in vitro in 3D. Primary melanoma cells were assembled into small tumors that were dispersed in a 3D matrix and challenged with patient-derived CIK or the NK-92 cell line. By means of imaging-based methods, we reported, visualized, and quantitatively measured the recruitment of CIK and NK on the 3D targets, their infiltration, and cytotoxic activity. Our results support the effective tumor recruitment and tumor infiltration by CIK and NK. Such features appeared dependent on the specific geometric aspects of the environment but can be explained in terms of directional migration toward the tumor, without invoking major feedback components. Overall, our 3D platform allows us to monitor the processes of tumor recruitment, infiltration, and killing by means of live measurements, revealing important kinetic aspects of ACT with CIK and NK against melanoma.

## 1. Introduction

Cancer immunotherapy is widely impacting and improving oncological treatments and patients’ outcome. Immune checkpoint inhibitors (CI), capable of overcoming tumor adaptive resistance and restoring effective T cell-mediated immune responses, currently dominate the therapeutic scenario [1,2,3,4,5]. Even acknowledging these important achievements, a relevant rate of patients still may not benefit from CI and refractory/relapsing diseases remain important issues and challenges for clinicians and researchers [1,6,7,8]. 

Cancer adoptive cell therapy (ACT) is emerging as an intriguing strategy, holding promises as an alternative or integrative approach in tumor settings poorly responding to CI. ACT relies on the ex vivo expansion/activation of anticancer lymphocytes to be infused in cancer patients. Multiple ACT strategies have been explored or are under investigation, exploiting either T lymphocytes or innate immune effectors. ACT approaches include pioneering tumor-infiltrating lymphocytes (TIL) in melanoma, more recent lymphocyte engineering by chimeric antigen receptors (CAR), and also promising data with Natural Killer (NK), γδT, and Cytokine-Induced killer (CIK) lymphocytes [9,10,11,12]. The great potential of these approaches is sustained by multiple preclinical evidence and initial clinical data [13,14,15,16]. 

In the last decades, since the first phase I trial in 1999 [17], several clinical trials supported the feasibility and safety profile of CIK antitumor activity in multiple tumor settings either as monotherapy or in combination with chemotherapies or even checkpoint inhibitors [18,19,20,21,22,23,24,25].

Currently there are 62 CIK studies registered on the official portal clinicaltrials.gov, including those not yet recruiting, active, or completed.

Translational research plays a crucial role in the exploration, evaluation, and comparisons of innovative ACT strategies, and reliable models are needed to promote their effective and rational transition into clinical studies. In this context, melanoma has been and continues to lead the field as an ideal experimental platform and clinical setting to explore innovative ACT [13,26]. 

Despite the promising results of ACTs, many of the fundamental biological and molecular mechanisms underlying the therapeutic success are not fully explored. In particular, the homing of immune cells and their interaction with the tumor into a complex microenvironment are yet to be totally understood and further research is needed to shed light on the many facets of this aspect of cancer [27,28].

Several in vitro experimental studies confirmed the antitumoral activity of immunotherapeutic ACT-based approaches. However, due to practical needs and simplicity of manipulation, many of the studies are limited to bidimensional culture assays [29,30]. While such culture conditions have undeniable experimental advantages, they tend to focus on interactions between immune and tumor cells, while disregarding the critical step of recruitment of immune cells on site. Three-dimensional (3D) cancer models represent a good alternative and integration to the traditional 2D cultures, without compromising the experimental needs. During the last decade, many studies were focused on the development of systems to recapitulate a realistic tumor environment. Cancer spheroids [31,32,33,34] and, more recently, patient-derived organoids [35], sometimes combined with techniques such as microfluidics or microfabrication, have promoted progress in the assessment of ACT-based therapies in 3D [36]. Three-dimensional models for cancer immunotherapy were recently reviewed in [31,32,37,38,39].

While the use of 3D cultures is standard routine and does not, itself, represent a challenging experimental technique, the coupling with cultures of antitumor lymphocytes and primary cancer cells might make it even more laborious and susceptible to the variability of all the components. This is particularly relevant in the case of the recruitment of immune cells by a tumor, where molecular diffusion and chemotaxis are involved [19], where timescales can easily span (at least) two orders of magnitudes, depending on the distance between effector and target. It is, therefore, useful, from the methodological point of view, to build models that can avoid unnecessary variability in geometry and culture conditions, where, on the other hand, the true heterogeneity (across different tumors or effectors) is preserved and not hidden. Such a model would empower the prospective clinical relevance of the observed experimental evidence, favoring the reliable exploitation of translational research for a more rational clinical trial design.

Here, we proposed a novel, 3D culture model as an experimental tool to explore the dynamic activity of ACTs against melanoma. In order to reduce the variability and the technical complications of the tumor microenvironment [40,41,42], we decided to focus on the interactions between immune cells and tumors by using a 3D collagen gel as a supporting scaffold. As tumor killer lymphocytes, we focused on either the NK cell line (NK-92) [43,44,45] or on patient-derived, cytokine-induced killer lymphocytes (CIK) [21,46,47]. We and others have previously reported the preclinical antitumor activity of both effectors by means of conventional 2D in vitro models, and their clinical efficacy is currently being explored in several clinical trials [14,48,49,50]. Here, the aim was to define a reliable model to investigate, quantify, and visualize such activity accounting for its dynamic components such as lymphocyte recruitment, tumor infiltration, and killing. In particular, the potential advantage of our 3D model is its portability to other effectors and tumors and its scalability to high throughput without compromising too much the need for quantitative approaches.

Both CIK and NK-92 cells are known to share a similar HLA-independent tumor-killing mechanism mediated, at least in part, by the recognition of stress-inducible molecules (MIC A/B; ULBPs) broadly expressed on tumor cells through their NKG2D receptor. Differently from NK, CIK cells still express their membrane T cell receptor, but it does not take part in the tumor-killing activity [51,52]. The use of HLA-independent effectors in the case of metastatic melanoma is motivated by the fact that, in clinical settings, the downregulation or alteration of HLA surface expression has been reported as a major tumor immune-escape mechanism [53]. Moreover, the HLA-independent tumor-recognition system is an advantage for our scope, as it allows reducing the variability in the antitumor cytotoxicity that may derive from TCR-mediated recognition of tumor-associated antigens and effective HLA antigen presentation [21,54,55]. The tumor setting of our study was that of advanced melanoma, and we exploited a previously developed bank of patient-derived melanoma cell lines.

## 2. Results

### 2.1. Modelling the Interactions between HLA-Independent Killer Lymphocytes and Melanoma Cells in 3D

**Figure 1:**
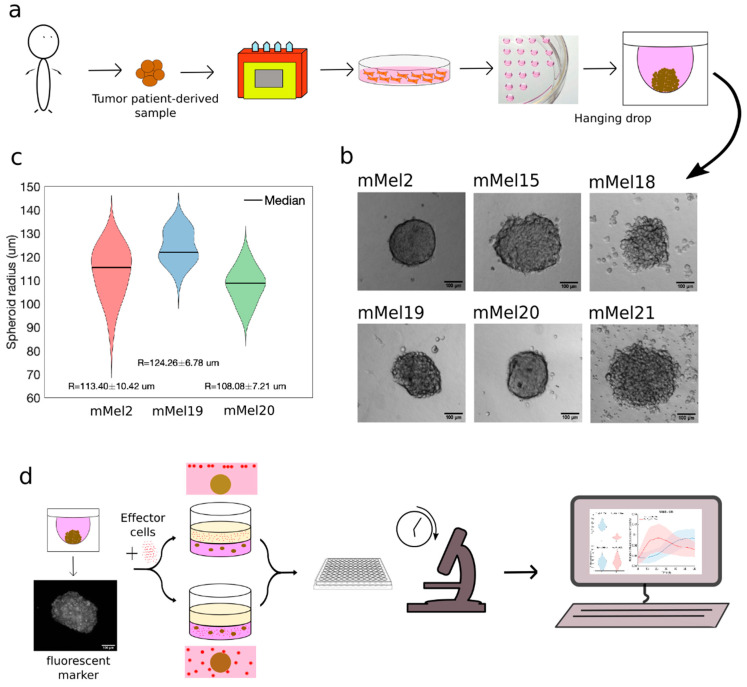
Establishment of a 3D model for the study of adoptive cell therapy against primary melanoma tumor samples. (**a**) Outline of spheroids formation. Melanoma patient-derived samples were processed to obtain primary cell lines. In order to obtain spheroids, cells were grown with the hanging drop technique. (**b**) We tested eight different primary melanoma samples for their ability in forming spheroids and successfully obtained spheroids from six of eight samples. Slightly different degree of compactness was observed, as expected, due to the heterogeneity of the different samples. (**c**) Violin plots indicate width and local density of the statistical distribution. Different colors refer to different samples, as indicated in the X tick. Numbers indicated report mean and standard deviation. (**d**) Sketch of the experimental procedure for the 3D embedding of target-effector co-cultures: Tumor spheroids and effectors were incubated with different fluorescent labeling agents and embedded in the appropriate ratio in a collagen gel (bottom) or spheroids were embedded and effectors added with the media on top of the gel (top). Imaging well plates were used to then monitor the assay with a motorized inverted microscope of choice. Digital images were post-processed for quantification and data analysis.

In order to dissect the different steps characterizing the interactions between killer lymphocytes and cancer cells in an adoptive cell therapy (ACT) setting, we set up a 3D co-culture method. To this aim, we exploited a previously established tumor sample biobank, which features cell lines obtained from eight surgical samples derived from metastatic melanoma. Patient-derived tumor samples were grown by means of the hanging drop technique [56] to form compact tumor spheroids starting from 2 × 10^3^ cells (Figure 1a). We successfully generated compact spheroids from six out of eight primary cell cultures (Figure 1b). Such procedure allowed us to obtain a series of tumor targets, uniform in size (Figure 1c), to be challenged with the activity of different antitumor lymphocytes. In order to reduce all the possible undesired sources of variability, we checked whether the spheroids obtained with our methods were sufficiently homogeneous in size and obtained relative dispersions of 17.7% for mMel2, 10.9% for mMel19, and 13.2% for mMel20. 

In order to mimic the 3D geometry typical of melanomas, tumor spheroids were embedded in a collagen gel, giving the necessary mechanical support to spheroids, yet allowing nutrient exchange and immune cell migration as well as the necessary practical advantages for cell culture. The desired number of spheroids (*n* = 10) was then placed in a 3D collagen gel, which was polymerized into a multiwell culture plate, forming a 3D tumor setting with melanoma spheroids (3D-Mel) (Figure 1d). 

To assess the ability of killer lymphocytes to reach and kill the different melanoma patient-derived samples, we added them on top of the collagen gel (Figure 1d, top) or alternatively mixed within the collagen matrix (Figure 1d, bottom). The first geometrical setting resembled more closely what might happen in vivo while the second allowed a more precise accounting for spatial distribution of lymphocytes. From the experimental viewpoint, the second choice had the advantage that single spheroids ideally represent separate experiments that can be compared, while the first was afflicted by the different distances of the spheroids from the initial position of the effectors. In order to be able to follow separate immune effectors and targets, we used live fluorescent transient markers and we performed custom-written image analysis to monitor the interactions between target and effector cells and to quantify antitumor therapy effect (Figure 1d). 

### 2.2. Recruitment of HLA-Independent Killer Lymphocytes toward Melanoma Target Cells in 3D

#### 2.2.1. Geometry Impacts the Recruitment of Tumor Killer Lymphocytes

The effective tumor recruitment of killer lymphocytes is a crucial, yet overlooked, step for ACT strategies and their translational models. To explore the rate and degree of tumor recruitment of killer lymphocytes we exploited our model coupled with optical imaging. We performed time-lapse experiments with 3D-Mel co-cultured with CIK or NK-92 cells by means of fluorescent excitation at different wavelengths and using brightfield as a reference.

**Figure 2:**
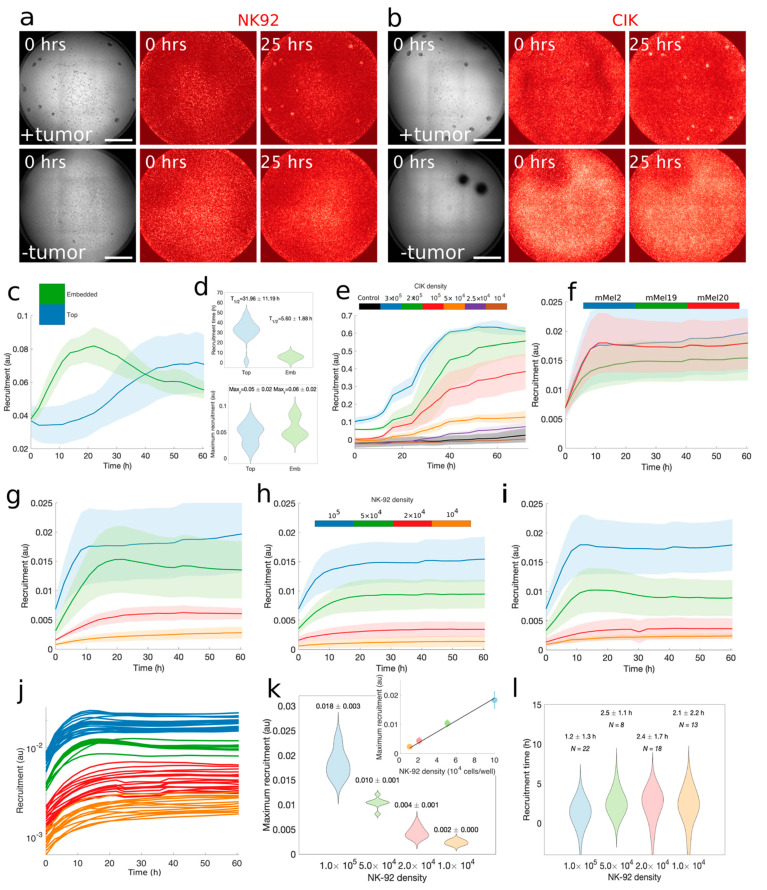
Homing of immune effector to target in 3D melanoma. (**a**,**b**) NK92 and CIK cells were embedded in collagen and were monitored by time-lapse imaging with (top rows) and without (bottom rows) target. The images correspond to two snapshots at indicated times for brightfield and fluorescent channels corresponding to excitation of PKH26, which labels effectors only. No clustering at the scale of target emerged. Scale bar: 500 μm. (**c**) CIK cells were embedded into a collagen matrix (green) or were seeded on top of the matrix (blue) and were monitored by time-lapse imaging. Recruitment in this and in the following panels and plots was measured as the intensity of fluorescence signal in the regions of the image corresponding to the spheroids. The plot shows the recruitment of effector cells on mMel19 spheroids at different times after the addition of CIK. Each line represents the average of many single spheroids (*N* = 14; *N* = 23 for top and gel seeding, respectively), while shades represent standard deviation. (**d**) Quantitative analysis obtained from recruitment data: (top) recruitment time extracted by fitting the recruitment time sequences as fluorescent signal localized onto the tumor spheroids for CIK seeded on top (blue) or mixed in the gel (green). Mean and dispersions were (31.96 ± 11.19) h for top-seeded CIK (*N* = 14 spheroids), compared with CIK cells embedded in the collagen (in green, (5.6 ± 1.88) h, *N* = 23 spheroids). Bottom: maximum recruitment of CIK cells on the target in terms of fluorescent signal on spheroids: (0.05 ± 0.02) when effector cells were seeded on top (blue) and (0.06 ± 0.02) when effector cells were embedded in collagen (*N* = 14; *N* = 23 for top vs. gel seeding, respectively). (**e**) Different concentrations of CIK cells were seeded on top of the collagen and let to recruit the tumors. Each colored line represents a density and is the average over three different wells for a total of many single spheroids (Black Ctrl *N* = 28; Blue 3 × 10^5^ CIK *N* = 36; Green 2 × 10^5^ CIK *N* = 28; Red 1 × 10^5^ CIK *N* = 29; Orange 5 × 10^4^ CIK *N* = 30; Violet 2.5 × 10^4^ CIK *N* = 30; Brown 1 × 10^4^ CIK *N* = 40), while shades indicate the standard deviation within the averages over each well. (**f**) The same number of NK-92 cells was mixed in the collagen gel for three different tumor samples: mMel2 (red), mMel19 (green), and mMel20 (blue). Each line represents the average over many single spheroids (*N* = 16, *N* = 16; *N* = 23, respectively) and shades indicate the standard deviation. (**g**–**i**) The mMel2, mMel19, and mMel20 were challenged with different densities of NK-92 (10^5^; 5 × 10^4^; 2 × 10^4^; 1 × 10^4^), indicated by the different colors (yellow, red, green, and blue, respectively). A significant increase of average fluorescence signal during the first 10 to 15 h and a persistent signal during the following hours was seen, correlating with the concentration of effector cells. Each line represents the average over several spheroids for the different densities, indicated here from larger to smaller densities: mMel2 *N* = 16, 11, 13, 19; mMel19 *N* = 6, 12, 19, 17; mMel20 *N* = 22, 8, 18, 13. Shades indicate the standard deviation. (**j**) The plot shows the single spheroids’ signals from which the averages in panel i were obtained. (**k**,**l**) Parameters of recruitment obtained by fitting the profiles in panel l. (**k**) Maximum recruitment of NK-92 cells on target: Higher concentration of effectors (1 × 10^5^ cells/well) showed a higher maximum recruitment, compared with lower concentration of effectors (1 × 10^4^ cells/well). The direct proportionality between recruitment and effector density is shown in the inset. (**l**) Time of recruitment for the four different densities of NK-92 cells: The distribution of recruitment times was comparable at different concentrations of NK-92 cells.

CIK or NK-92 cells were embedded in collagen with and without melanoma target, with the aim of excluding their spontaneous clustering on the scale of the tumor size. Spontaneous effector clustering did not occur on a spatial scale of more than 10 μm, while recruitment of CIK and NK-92 on target appeared on the scale of the size of the spheroid (i.e., around above 100 μm) (Supplementary Materials: Videos S1, S2 and S3). These results ruled out the emergence of large-scale, high-density patches or the mass migration of CIK and NK-92 in some defined regions of the culture vessel, validating the use of fluorescence intensity as a faithful proxy for recruitment (Figure 2a,b). 

Next, we inquired about the difference in kinetics if killer lymphocytes were added on the liquid–gel interface or embedded within the gel. To this aim, we measured the recruitment of killer lymphocytes on target as a function of time in two parallel experiments, as shown in Figure 2c. To extract quantitative information from the shape of the curves, we fitted recruitment profiles with a sigmoid function and we obtained four parameters: baseline and maximum recruitment, recruitment time, and rate. Details are given in the Materials and Methods section. Recruitment of CIK seeded on top took on average (30.4 ± 0.7) h while for matrix embedded CIK it took only (5.9 ± 0.8) h, with a relative delay of more than a day. The recruitment speed was also significantly affected by effector position into gel, with top-seeded CIK having a rate of (0.16 ± 0.02) $h^{-1}$, while almost twice that rate (0.29 ± 0.05) $h^{-1}$ was observed for matrix-embedded ones. We also noticed a larger dispersion of the distribution of times (Figure 2d, top), due to the different distance of CIK from target: 11.19 vs. 1.8 when seeded on top or embedded into gel, respectively. However, we did not observe significant differences between the overall fluorescent signals, meaning that despite the distance, a comparable number of CIK was recruited on all spheroids (Figure 2d, bottom), even though the process took longer times overall. This was corroborated by the fact that adding more lymphocytes on top resulted in a higher recruitment, as measured by the increase in fluorescence, as shown in Figure 2e. 

While CIK were able to reach their target in both settings (seeded on top or embedded), in this particular setting NK-92 were slower or ineffective when seeded on top of the gel (Supplementary Materials: Figure S1c). This result validated the 3D-mel as a simple method able to detect different rates and effective recruitment abilities of different types of killer lymphocytes.

#### 2.2.2. Recruitment toward Distinct 3D Melanoma Targets.

In order to increase the throughput of our assay, we imaged several tumor spheroids from three distinct primary melanoma cell lines, as representative of different melanoma conditions: BRAFV^600^ mutated (mMel2) and wild type (mMel19, mMel20). Spheroids were mixed with NK-92 in a collagen matrix. We first wondered whether we could observe a similar recruitment for the same number of killer lymphocytes targeting different samples. We found only minor differences in the time of recruitment (2.4 h, 1.8 h and 3.3 h for mMel2, mMel19 and mMel20 respectively) and maximum recruitment (0.016, 0.012 and 0.014, for mMel2, mMel19 and mMel20 respectively) as shown in Figure 2f. Rates were also comparable. This observation suggests that variability in the process of killer lymphocytes recruitment against multiple melanoma spheroids is relatively low.

To assess the rate and degree of lymphocyte recruitment as a function of time and density we added increasing concentrations of NK-92 cells, measuring the average fluorescence intensity on spheroids in a time-lapse (Figure 2g–i). At the beginning of the experiment, the baseline fluorescence signal corresponding to spheroids was 2 to 5-fold higher in 3D-Mel that included lymphocytes, compared with control, due to the interactions between cells taking place during the preparation of the experiment (Supplementary Materials: Figure S1d). In the following hours, the recruitment actively took place during the first 10 to 15 h of the assay, while we found the signal to be persistent for the following 45 h. For all melanoma lines tested there was a clear proportionality between the NK-92 recruitment and their amount seeded, suggesting the absence of a strong feedback on the amount of lymphocyte recruitment.

To further verify this concept, we considered each single tumor spheroid in our assays as independent and performed measurement of recruitment on each separately, by using a fit to a sigmoid (see Materials and Methods). The single spheroid curves are shown in Figure 2j. The detailed analysis of maximum lymphocyte recruitment confirmed a direct proportionality between lymphocyte recruitment and their density (Figure 2k), but not between lymphocytes density and recruitment time. The distribution of the recruitment time is similar at different NK-92 density. This indeed is consistent with a migration speed that is largely independent of density (Figure 2l).

### 2.3. Infiltration of HLA-Independent Killer Lymphocytes into 3D Melanoma Spheroids

**Figure 3:**
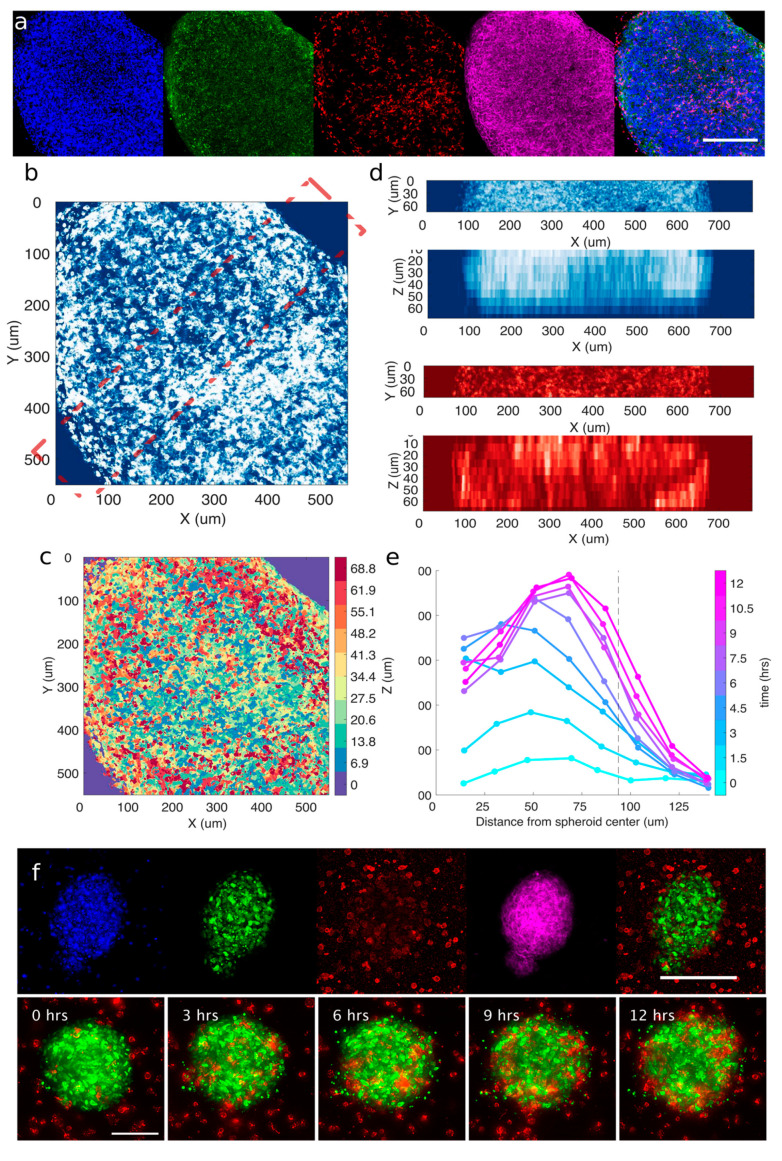
Effectors are infiltrating the tumor masses. (**a**) Representative single confocal section of a mMel2 spheroid challenged with CIK after 70 h. (blue: dapi; green: anti-caspase 3; red: CD45; magenta: phalloidin; mixed: merge of dapi, CD45, and caspase). Scale bar: 200 μm. (**b**) Maximum intensity projection of confocal sections from data set shown in panel (**a**). False colors go from low intensity (dark blue) to high intensity (white). (**c**) Maximum intensity projection of confocal sections from data set shown in panel (**a**), with color coding corresponding to height of each pixel. Color bar on the right of the image shows the different colors used to identify planes from the stack. (**d**) Projections of data set shown in panel (**a**), sliced according to the dashed, red mark shown in panel (**b**). Top (bottom) rows correspond to xy (xz) maximum intensity projection for dapi signal (white on blue) or CD45 (white on red). (**e**) Radial profiles of recruitment (fluorescence intensity) were obtained by averaging radially the fluorescence signal. Each line represents a time, as indicated in the color bar. The dashed, black line represents the boundary of the spheroid. (**f**) Top: representative, single confocal section of a M005 spheroid challenged with NK-92 after 50 h. (blue: dapi; green: H2B-GFP; red: CD45; magenta: phalloidin; mixed: merge of H2B-GFP and CD45). Scale bar: 200 μm. Bottom: time sequence of a time-lapse experiment with mMel2-H2BGFP (green) and NK-92 (red). Images are maximum intensity projections of deconvolved images. Scale bar: 100 μm.

To understand if the cytotoxic effect of ACT is carried out by means of effector–target interactions on the surface of the tumor or whether effectors are able to penetrate the mass, we fixed and immuno-stained Mel spheroids during our 3D co-culture essay with CIK. Fixed 3D samples were then imaged by means of a confocal microscope (Figure 3a). Immunostaining with anti-caspase 3 antibody allowed us to immediately see that apoptotic events were not restricted on the surface of the spheroids, hinting at the fact that toxicity induced by killer lymphocytes might not be limited to the surface of the tumor. To corroborate this observation, we performed several visualizations of 3D data from a confocal z-stack in order to assess the positioning of the effectors in the spheroid. Even in the case of large spheroids, CIK can be found at least a few layers within the tumor (Figure 3b–d). This supports the hypothesis that cytotoxic activity is not fully restricted to the surface but also occurs within the tumor. 

**Figure 4:**
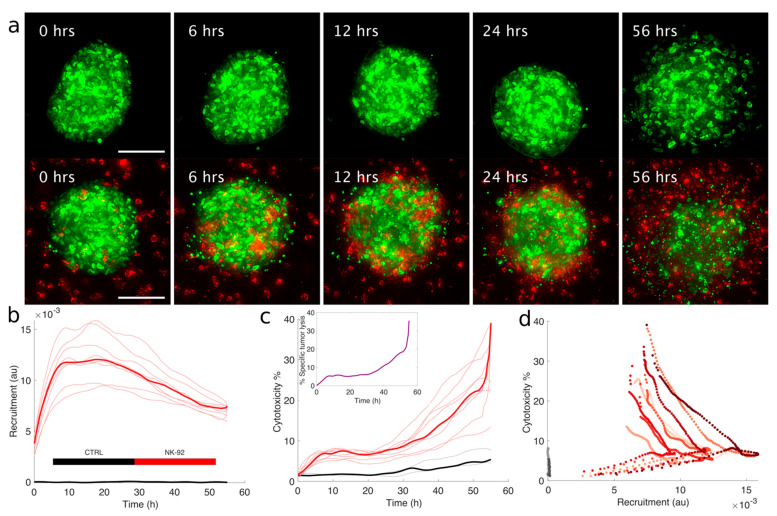
Quantitative characterization of tumor-specific cytotoxic activity of lymphocytes in 3D Mel. (**a**) Snapshots of mMel2-H2B-GFP spheroids alone (top) or co-cultured with NK-92 cells in 3D (bottom). Green signal corresponds to H2B-GFP while red signal comes from PKH26 labeling. Images are maximum intensity projections of a deconvolved z-stack time lapse. Scale bar: 100 μm. (**b**) The plot illustrates the recruitment of NK-92 cells on mMel2 spheroids as reported by the fluorescence signal corresponding to single spheroids over time in control (black) vs. treated with NK-92 (red). Grey and light red correspond to single spheroids, while black and bright red correspond to averages. (**c**) Cytotoxicity for mMel2 left untreated (black) or co-cultured with NK-92 (red) as a function of time, calculated as the fraction of fragmented nuclei over the total amount of nuclei in each image, digitally segmented. Grey and light red correspond to single spheroids, while black and bright red correspond to averages. The inset shows a surrogate of the tumor-specific killing, calculated by assuming that fully eradicated tumors correspond to 100% of nuclei fragmented. (**d**) Scatterplot of recruitment vs. cytotoxicity. These curves are V-shaped because recruitment tends to be a non-monotonic function of time, while the curve for cytotoxicity is. This indicates that the bulk of the cytotoxic effect is not synchronous with the beginning of recruitment. Shades of grey and red correspond to the different spheroids plotted in panels (**b**,**c**).

We then wondered whether we could observe killer lymphocytes penetrating the target dynamically. Therefore, in order to have a more stable nuclear marker, suitable for long-term imaging with 3D optical sectioning, we employed a lentiviral vector coding for histone 2B conjugated with a fluorescent protein (H2B-GFP). Spheroids of mMel2-H2B-GFP cells were embedded in collagen with NK-92 cells and were imaged over time, as shown in a representative snapshot in Figure 3f and Figure 4a. Single spheroids were monitored by time-lapse fluorescence microscopy in order to measure the position of effector cells with respect to the whole spheroid. Monitoring the fluorescence level in a section of the spheroid reveals that NK-92 recruitment occurs even several cell layers within the spheroid as soon as 3 h after the beginning of the experiment (Figure 3e). The vast majority of lymphocytes were, however, recruited on outer shells of the tumor and persisted over several tens of hours. 

### 2.4. Cytotoxic Activity by HLA-Independent Killer Lymphocytes in 3D-Mel Spheroids

#### 2.4.1. Evaluation of Cytotoxic Activity with High-Resolution 3D Live Imaging Assay 

As the last step, we aimed at exploring the cytotoxic activity of CIK and NK-92 within the context of 3D-Mel. A technical and practical challenge for the functional evaluation of tumor-killing activity is represented by 3D geometry. To overcome this technical complication, we employed a method based on the measurement of nuclear fragmentation as a proxy for cytotoxicity [57] and we followed mMel2-H2B GFP and lymphocytes with high spatio-temporal resolution, as shown in Figure 4a and in Supplementary Materials: Videos S4 and S5.

Labeled NK-92, mixed within the collagen gel, were effectively recruited on the mMel2-H2B GFP spheroids in a few hours, with the maximum signal of recruitment occurring between 10 and 25 h (Figure 4b). We then performed digital segmentation (see Materials and Methods) and identified the fraction of intact and fragmented nuclei within the spheroid and used the relative fraction of fragmented nuclei over the total as a proxy for cytotoxicity. The fraction of intact nuclei correspondingly decreased for treated samples, while it was stable over time for untreated samples. The data indicated that the cytotoxic activity of NK-92 was progressively increasing during recruitment, with a specific tumor lysis around 10% after 40 h, 40% at 56 h of treatment, and progressive fragmentation of the whole spheroid and, thus, almost complete eradication was observed toward the end of the experiment (Figure 4c). From the simultaneous assessment of both recruitment and cytotoxic activity, it was clear that a substantial tumor-killing activity can occur only after a significant lymphocyte recruitment, as shown in Figure 4d. 

#### 2.4.2. Cytotoxic Activity with Higher Throughput 3D Live Imaging Assay 

**Figure 5:**
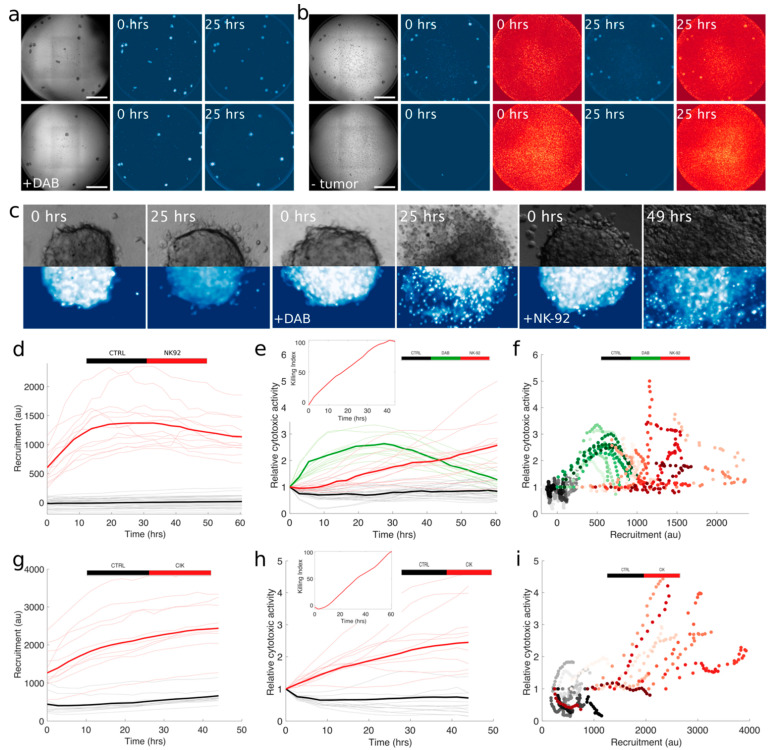
High-content analysis of ACT of tumor-specific cytotoxic activity of lymphocytes in 3D Mel. (**a**,**b**) Representative images of mMel2 spheroids cultured in multiwell plates with tumor spheroids labeled with NucBlue (blue colormap) and effectors labeled with PKH26 (red colormap). Grey pictures correspond to brightfield images. Images reporting the label +DAB correspond to mMel2 spheroids treated with 2.5 µM of Dabrafenib. Scale bar: 500 μm. (**c**) The images represent control spheroids, spheroids treated with dabrafenib, and with NK-92 at two different times of the experiments. The top half of the image is the bright field channel while the lower part corresponds to tumors labeled with NucBlue. (**d**) Recruitment, corresponding to high-content imaging of mMel2 tumor spheroids, both untreated (black) and co-cultured with NK-92 (red). Light hues correspond to single spheroids (*N* = 30 for ctrl, *N* = 13 for NK-92), while bright colors correspond to averages. (**e**,**f**) Cytotoxic activity and scatter plot corresponding to high-content imaging of mMel2 tumor spheroids either untreated (black), co-cultured with NK-92 (red), or treated with Dabrafenib (green). Light hues correspond to single spheroids (*N* = 30, 11, and 13 for ctrl, DAB, and NK-92, respectively), while bright colors correspond to averages. (**g**–**i**) Recruitment, cytotoxic activity, and scatter plot, corresponding to high-content imaging of mMel2 tumor spheroids either untreated (black) or co-cultured with CIK (red). Light hues correspond to single spheroids (*N* = 15 and 12 for ctrl and CIK, respectively), while bright colors correspond to averages.

To verify whether we could extend our approach to a higher throughput we seeded 10 spheroids per well on a multiwell plate and extended our fragmentation-based method on samples imaged with lower magnification, in order to increase the speed of our imaging steps and the representativity of our sample, at the price of image resolution. Well-wide imaging was performed in order to monitor several tumor spheroids at the same time while increasing greatly the throughput of the assay. Cultures were imaged for 70 h by time-lapse fluorescence microscopy. Spheroids were segmented, thanks to brightfield images, while effectors and nuclear fragmentation in the target were followed independently by fluorescent labeling with live dyes, as shown in Figure 5a,b. This approach was made possible with the technical setup of cytotoxicity assays presented in Section 2.3 and Section 2.4.1, and it also allows a broader applicability because it does not require a genetic modification of the tumor samples. The technical validity of the assay was first confirmed by treatment with BRAFi Dabrafenib, active against MEL-spheroid (mMel2) harboring the target BRAF mutation. The effect of the treatment was verified by means of an independent technical approach, as shown in Figure S1f. The effect of Dabrafenib appears rapidly, as nuclear fragmentation is visible within the first 3 h of treatment, and its effect is complete within 24 h, as shown in Figure 5c. We were able to apply nuclear fragmentation methods to multiple spheroids simultaneously with both NK-92 and CIK, with results that were qualitatively consistent with those obtained with high-resolution images. Cytotoxic activity of NK-92 increased slowly during the first 10–20 h, corresponding to the recruitment time (Figure 5d). After the increase of recruitment in 20–30 h, we observed a progressive increase of specific tumor lysis, with the amount of fragmented nuclei increasing about 2.5 fold in 60 h, compared to the baseline fragmentation observed in the control (Figure 5e). We could not register the significant acceleration observed with higher-resolution experiment due to the fluorescent signal of the live marker fading out after 3 days of culture. Analogously, we could monitor the recruitment and, simultaneously, the cytotoxic activity of CIK (Figure 5g,h), and obtained similar results in terms of kinetics. This version of the assay allowed, once more, the concomitant evaluation of killer lymphocytes’ recruitment and cytotoxic activity (Figure 5f,i) and recapitulated qualitatively the results obtained with the higher-resolution method coupled with the fluorescent protein.

## 3. Materials and Methods

### 3.1. Patient-Derived Melanoma Cell Lines

Melanoma cell lines (mMel2, mMel3, mMel7, mMel15, mMel18, mMel19, mMel20, mMel21) were generated in our laboratory from patient-derived, fresh, surgical biopsies of advanced melanoma [30,58]. The collection of patients’ biological samples and the informed consent were approved by the internal review board (Prot. Number 225/2015). Patient-derived mMel were cultured in KO DMEM F12 (KO Out Dulbecco’s Modified Eagle Medium, Gibco BRL, Thermofisher, Waltham, MA, USA), with 10% FBS, 25 mmol/L HEPES, 100 U/mL penicillin, and 100 U/mL streptomycin (Gibco BRL, ThermoFisher, Waltham, MA, USA) in a humidified 5% CO_2_ incubator at 37 °C. The cell lines obtained were characterized by flow cytometry (CyAn ADP, Beckman Coulter, Brea, CA, USA): All primary cell lines were found positive for HLA-ABC, β2 m, and NKG2D ligands (MIC A/B; ULBP2,5,6). 

### 3.2. CIK Culture and Expansion

CIK were generated in vitro from peripheral blood mononuclear cells (PBMC) isolated from patients with histologically confirmed melanoma. The collection of patients’ biological samples and the informed consent were approved by the internal review board (Prot. Number 225/2015). PBMCs were isolated and collected from blood by density gradient centrifugation using cell-separation media (Lymphosep, Aurogene, Roma, Italy). PBMCs were cultured overnight in cell-culture flasks at a cell density of 2 × 10^6^/mL in RPMI medium (Sigma-Aldrich, St. Louis, MO, USA) supplemented with 10% fetal bovine serum (Gibco BRL, ThermoFisher, Waltham, MA, USA ), 2 mM L-glutamine (Sigma-Aldrich, St. Louis, MO, USA), 1% PenStrep (100 U/mL Penicillin and 100 μg/mL Streptomycin, Sigma-Aldrich St. Louis, MO, USA), and 1000 U/mL IFN-γ (Miltenyi Biotec, Bergisch Gladbach, Germany). After 24 h in culture at 37 °C and 5% CO_2_, 50 ng/mL anti-CD3 antibody (MACS GMP CD3 pure, Miltenyi Biotec, Bergisch Gladbach, Germany) and 300 U/mL recombinant human IL-2 (Miltenyi Biotec, Bergisch Gladbach, Germany) were added. Fresh medium with IL-2 was added as needed. A weekly phenotypic analysis of CIK cells was performed, using fluorescein isothiocyanate (FITC), phycoerythrin (PE), or allophycocyanin (APC)-conjugated mouse mAbs: CD3- FITC, CD8-PE, CD56-APC, and CD314-APC, anti-NKG2D (Miltenyi Biotec Srl, Bergisch Gladbach, Germany) and anti-DNAM-1 (BD Pharmingen, San Diego, CA, USA). Labeled cells were read on FACS Cyan (CyAn ADP, Beckman Coulter, Brea, CA, USA) and analyzed using Summit Software (Beckman Coulter, Brea, CA, USA). Gate criteria were set to isotype controls (representative plots of CIK phenotype is reported in Supplementary Materials: Figure S1a).

### 3.3. NK-92 Cell Line 

NK-92 cell line was provided by DSMZ cell bank. NK-92 were seeded in cell culture flasks at a concentration of 2 × 10^5^ cells/mL in Alpha Minimum Essential medium (α-MEM, Sigma-Aldrich, St. Louis, MO, USA) without ribonucleosides and deoxyribonucleosides with the addition of 2 mM L-glutamine, 1% PenStrep (100 U/mL Penicillin and 100 μg/mL Streptomycin, Sigma-Aldrich, St. Louis, MO, USA), 12.5% fetal bovine serum (Gibco, Thermofisher, Waltham, MA, USA), and 12.5% horse serum (Gibco, Thermofisher, Waltham, MA, USA ). NK-92 cells were characterized by flow cytometry (Cyan ADP, Beckman Coulter, Brea, CA, USA) for the following receptors: CD3, typically expressed on T lymphocytes but absent on NK; CD56 (NCAM receptor), highly expressed on NK cells; NKG2D (Natural Killer Group 2D receptor) and NKp (natural cytotoxicity receptors)-30 and -46, involved during the NK activation; and PD-1 and TIM-3, involved during the NK inhibition (Supplementary Materials: Figure S1b). 

### 3.4. Spheroid’s Formation and Co-Cultures in Collagen Matrix

In order to have compact tumor cell aggregates (patient derived spheroids), cells from confluent primary cultures were detached with 0.05% trypsin EDTA (Thermofisher, Waltham, MA, USA) and the spheroid formation was performed using the hanging drop technique [56]. Cells were resuspended in full Knock Out DMEM F12 medium (KO Out Dulbecco’s Modified Eagle Medium, Gibco BRL, Thermofisher, Waltham, MA, USA) supplemented with 2.4 mg/mL of methylcellulose (Sigma-Aldrich, St. Louis, MO, USA), at the final concentration of 5 × 10^5^ cells/mL. Then, 40-μL droplets containing 2000 cells were pipetted onto an inverted lid of a petri dish and incubated up to 48 h at 37 °C and 5% CO_2_. Subsequently, spheroids were collected, washed with phosphate buffered saline (PBS, Sigma-Aldrich, St. Louis, MO, USA), and were embedded in collagen. Melanoma spheroids were suspended in a solution consisting of 1.5 mg/mL collagen rat tail type I (Corning, New York, NY, USA), 15 mM NaOH, and 30 mM HEPES 1M (Sigma-Aldrich, St. Louis, MO, USA) in RPMI medium (Sigma-Aldrich, St. Louis, MO, USA). In some cases, based on the experiment performed, lymphocytes’ cells (CIK or NK-92 cells) were added into the collagen solution at densities varying between 2 × 10^5^ cells/mL and 4 × 10^6^ cells/mL. Differently from 2D assays, in the 3D case the effective number of effector/target ratio was expressed in terms of volume densities rather than absolute numbers. The number of lymphocytes to be seeded in the gel was estimated by calculating the density of effector cells that could fit in a spherical shell (300 μm radius) surrounding each tumor spheroid, in order to approximately generate a theoretical 1:1 effector/target ratio around all spheroids. The mix was pipetted into a black-walled, glass-bottom, 96-well imaging plate (Eppendorf) (pre-coated with the same collagen solution) and was allowed to polymerize for 30–40 min in the incubator at 37 °C, 5% CO_2_. After polymerization, the scaffolds were covered with DMEM-F12 w/o phenol red medium (Thermofisher, Waltham, MA, USA).

### 3.5. Immunostaining and Confocal Microscopy

Immunofluorescence was performed according to standard protocols. Briefly, 3D cultures were grown in 8-well Chamber Slide with removable wells (ThermoFisher, Waltham, MA, USA). After 72 h, cells were fixed in 4% (*w*/*v*) paraformaldehyde in PBS for 30 min at 4 °C and were permeabilized with 0.5% (*w*/*v*) Triton X-100 in PBS for 1 h at room temperature. After 1 h of incubation with blocking solution (0.2% (*w*/*v*) Triton X-100 and 5% Donkey serum (Sigma-Aldrich, St. Louis, MO, USA), the cultures were incubated with the primary antibody (anti-CD45, 1:100, Dako, Agilent, Santa Clara, CA, USA), re-suspended in the blocking solution, and kept overnight at 4 °C. The following day, cultures were washed with PBS (×3) and incubated with the secondary antibody (anti-mouse 555, 1:400, Invitrogen™, ThermoFisher, Waltham, MA, USA), DAPI (1:1000, Roche, Basel, Switzerland), and Alexa Fluor 647 Phalloidin (Invitrogen™, ThermoFisher, Waltham, MA, USA), in blocking solution for 1 h at RT and rinsed with PBS (×3). Images were acquired with a confocal microscope (Leica SPEII, Leica microsystems, Wetzlar, Germany) with a 20× objective and processed with Matlab (The Mathworks, Natick, MA, USA). 

### 3.6. Melanoma Primary Cells’ Labeling

We employed imaging-based methods to describe experimentally the effect of a given ACT on a particular tumor sample and to extract quantitative measurements. In order to be able to recognize effector and target cells and to dynamically monitor recruitment and the efficacy of effector cells, we employed commercially available no genetic transient fluorescent dyes (PKH26 from Sigma-Aldrich for CIK and NK-92 and NucBlue from Invitrogen for melanoma cells) and lentiviral infection with a nuclear protein tagged with a fluorescent protein (H2B-GFP). This double approach allowed us to cover the different needs for space and time resolution of optical imaging and face the potential issues coming from either approach. By making use of imaging glass-bottom plates and combining such labeling techniques, we were able to image target and effector plates with different space and time resolution, depending on the experimental question, as detailed in what follows. 

The lentiviral vector LV-GFP used for transduction was a gift from Elaine Fuchs (Addgene plasmid #25999). Melanoma primary cells (mMel2) were re-suspended in full KO DMEM-F12 medium and viral preparation was added at a dose of 100 ng P24/1 × 10^5^ cells, with a final concentration of 4 µg/mL polybrene. After 24 h, cells were washed and grown for 10 days with fresh medium. In order to confirm the GFP expression and the efficiency of transduction, we performed flow cytometry analysis. 

As a way to visualize interaction between spheroids and lymphocytes in time-lapse imaging, cells were stained with different labels before performing the hanging drop technique and setting up the experiments. Melanoma cells were stained with nuclear live staining NucBlue (Invitrogen™, ThermoFisher, Waltham, MA, USA), while CIK cells and NK-92 cells were stained with live dye PKH26 (Sigma Aldrich), following the manufacturer’s instructions. Melanoma spheroids were mixed with collagen and lymphocyte cells and were seeded on a coat of collagen in black-walled, glass-bottom, 96-well imaging plates, as described above. Imaging was performed either on a cell imaging multi-mode microplate reader (Cytation3, BioTek, Winooski, VT, USA) with a 4× objective or with an inverted widefield microscope Nikon Ti2 (Nikon Instruments, Melville, New York, NY, USA) with a 40× dry objective, equipped with a z-motor in order to acquire z-stacks. Images were processed either with Nikon NIS Element (Nikon Instruments) or with custom-written Matlab (The Mathworks) scripts.

### 3.7. Image Processing and Data Analysis 

Spheroids were segmented using the brightfield channel and training the machine learning software Ilastik [59]. Binary masks obtained with segmentation were used to calculate the recruitment as the mean fluorescence in the red channel with background subtraction, calculated in the regions of the image with no spheroids. From a methodological point of view, rather than employing a specific apoptosis detection kit, we used fragmentation of nuclei as a proxy for apoptosis. This choice was motivated by the complication of separating cell death of target from that of effectors, by the need of keeping the amount of light on the sample low, and by the potential need of having at least a semi-quantitative measurement. To this aim, we set up a dedicated quantitative image analysis pipeline to detect fragmentation of nuclei and employed two separate approaches, depending on the resolution of the images. High-resolution images were fed into Ilastik in order to separate intact from fragmented nuclei regions. The ratio between fragmented and total nuclear regions was used as a measure of relative fragmentation. For low-resolution images, image sharpening was performed in order to enhance apoptotic bodies’ signal. The processed image was then thresholded and the fraction of positive spheroids (with respect to the total spheroid area, identified with segmentation, as explained above) was used as a proxy for nuclear fragmentation. All image analysis steps were performed, unless noted otherwise, with Matlab. 

Recruitment profiles were fitted with a generic sigmoid function of the form $y=a+b\frac{exp\left(k\left(t-\tau \right)\right)}{1+exp\left(k\left(t-\tau \right)\right)}$ where parameters *a* and *b* represent the baseline and maximum recruitment, respectively, while k is the recruitment rate and τ is the recruitment time (delay). Profiles were fitted with Matlab. 

A word of caution is appropriate for the estimation of the tumor-specific killing or lysis. The proxy we used for the cytotoxic activity was the surface of a 2D image occupied by fragmented nuclei divided by the total surface occupied by the spheroid (i.e., intact plus fragmented nuclei). It should be noted that this quantity, ignoring issues related to the 2D projection of a 3D object for the sake of simplicity, is not, strictly speaking, proportional to the number of dead cells unless the signal is particularly stable overtime. Another possibility is to have signals that are extremely labile overtime and estimating the number of apoptotic cells by counting the number of events. However, control experiments with Dabrafenib tell us that the stability of the signal has a timescale of the order of roughly 24 h. Therefore, dead cells start appearing as bright dots but disappear over time. This fact makes the precise counting of dying cells impossible and allows only rough estimates. To independently test the induction of apoptosis we used the commercially available fluorescent marker CellEvent Caspase3/7 (C10423, Thermofisher, Waltham, MA, USA), used according to the manufacturer instructions, at a concentration of 2 µM.

## 4. Discussion 

In this work we successfully generated a 3D experimental adoptive immunotherapy model to dissect the interactions between melanoma tumor cells and antitumor lymphocytes. Our approach addressed one of the paradigms of adoptive immunotherapy in solid tumors, i.e., the decomposition of the action of immune effectors into different interconnected aspects: effectors’ recruitment to the target, effective tumor mass infiltration, and cytotoxic capability. It has to be noted that while these are logically separated steps, they cannot be considered temporally separated or sequential at a bulk level. Even in our pre-clinical setting, all these steps occurred simultaneously and, therefore, the theoretical scheme of considering them as separate phenomena should be taken as a simplified framework. 

As an ACT model, we focused on an HLA-independent approach exploiting two distinct immune effector types, sharing the same NKG2D-mediated antitumor mechanism. This choice was dependent on our previous preclinical experience with such killer lymphocytes within 2D models in various tumor settings, along with the promise they hold for clinical applications.

This study partly overcame the evident limits of studying lymphocyte homing and melanoma infiltration in a two-dimensional assay. Two-dimensional assays, indeed, are devoid of a surrounding environment and tend to put targets and effectors into contact directly, skipping completely the recruitment problem. In this respect, the collagen matrix in the 3D-mel represented a controlled and experimentally practical support to melanoma cells, allowing lymphocyte migration and a more geometrically realistic tumor setting. The antitumor activity of ACT in solid tumors was, indeed, not exclusively restricted to the direct contact-mediated killing of tumor cells. It was, rather, composed of a whole cascade of functionally relevant events leading to the final step of tumor killing. The advantage of our 3D-mel model consists in the possibility to simultaneously investigate the homing, infiltration, and cytotoxic activity of killer lymphocytes toward melanoma spheroids. Thanks to the employment of imaging methods, our approach was amenable to quantitative and real-time observations that allowed precise kinetic characterization of the biological phenomena at play. Furthermore, our approach can be, in principle, extended to any other solid tumor that is able to grow in a hydrogel and that forms multicellular aggregates. 

The aim of evaluating in a reproducible manner the kinetic of recruitment of tumor killer lymphocytes to the target was one of the main points of the current paper. In a realistic in vivo setting, different, spatially separated masses might possibly exist, implicating heterogeneities of response of different kinds, from biological to physical, and it would be, therefore, useful to build a model where these aspects can be addressed. On the other hand, such variables become an obstacle to the experimental need of reproducibility when comparing different tumors or distinct therapeutic approaches. In this view, it is, therefore, important to limit most types of variability. This observation was made evident by the measurements of the time of recruitment. Killer lymphocytes were placed on the top of the matrix gel to resemble more closely what might happen in vivo, where effector cells might have to traverse collectively a layer of tissue or stroma, due to systemic or local infusion. In this situation, indeed, since the position of the spheroids in the gel was not predefined, the outcome of killer lymphocytes’ activity on different targets resulted in being very heterogeneous and hard to compare, as the variability given by the spatial distribution of the tumor spheroids was high. 

Another issue that can be faced by the approach we proposed is related to the migration velocity of antitumor lymphocytes, their sensitivity to the chemotactic cues, and the range of sensitivity. Our 3D model is amenable to such quantitative evaluation, even to experimentally discriminate and compare the ability of different killer lymphocytes, commonly used in ACT approaches. Our preclinical model is, indeed, capable to evaluate and compare the different lymphocyte recruitment capability by different melanoma, accounting for potential tumor intrinsic variables and potentially allowing a more precise comparison of ACT approaches that might potentially be considered in different tumor contexts. 

We observed a specific recruitment on three different melanoma samples and found that the number of immune effectors recruited on tumor spheroids was proportional to the number of effectors added in the medium. We confirmed the direct proportionality between amount of recruitment and lymphocytes’ density, suggesting that no apparent strong saturation effect (i.e., too many immune effectors impair the recruitment) or feedback mechanism (i.e., more effectors induce a stronger recruitment) seem to be at work. In support of this observation, the kinetics of recruitment was found to be largely independent of the density of immune effectors, indicating that the velocity of recruitment is not affected significantly by density. Our findings were generated from a reliable, patient-derived, preclinical model and might provide clinically relevant insights helpful in the rational design of doses and schedules for ACT clinical studies. It is, however, important to acknowledge that, even if derived in origin from surgical biopsies, our tumor samples have become cell lines after multiple passages and dedicated experiments with freshly derived tumor samples may be warranted as more informative.

It would be interesting to enlarge the cohort of tumor samples in order to verify whether significant deviations from the response observed and reported here can occur and, ultimately, what are the possible underlying molecular mechanisms. 

A significant step in the success of ACT is the infiltration of lymphocytes into tumors. The kinetics of an ACT approach, especially for large masses, might be significantly affected by an efficient infiltration. It should be noted that the lymphocyte tumor infiltration model was artificial, as it considered a pre-formed tumor that was able to freely grow and then at a given moment was exposed to killer lymphocytes. While this scenario is a useful playground for the study of how recruitment might impact the kinetics of tumor killing, the impact of tumor size on the recruitment, infiltration, and killing by immune effectors should be more deeply investigated. Starting from our findings, the presented model is amenable to structural and functional implementations, like the addition of cellular and stromal complexity with the aim of a progressively increasing resemblance of the realistic tumor microenvironment.

## 5. Conclusions

In conclusion, our work represents an experimental platform to quantitatively investigate key aspects of tumor ACT and paves the way for future efforts in understanding the mechanistic basis of the biological interactions between immune effector cells and tumor spheroids, which are critical for a rational optimization of adoptive cell therapy approaches.

## Data Availability

The raw experimental data presented in this study are available on request from the corresponding author.

## Supplementary Materials

The following are available online at https://www.mdpi.com/article/10.3390/cancers13102302/s1. Figure S1: Supplementary Figure. Videos S1–S3: Dynamic representation of CIK and NK-92 recruitment toward melanoma targets. Video S4 and S5: Recruitment and cytotoxicity of NK-92 and CIK.

## References

[1] Darvin P., Toor S.M., Sasidharan Nair V., Elkord E. (2018). Immune checkpoint inhibitors: Recent progress and potential biomarkers. Exp. Mol. Med..

[2] Queirolo P., Spagnolo F., Ascierto P.A., Simeone E., Marchetti P., Scoppola A., Del Vecchio M., Di Guardo L., Maio M., Di Giacomo A.M. (2014). Efficacy and safety of ipilimumab in patients with advanced melanoma and brain metastases. J. Neuro Oncol..

[3] Feng Y., Roy A., Masson E., Chen T.T., Humphrey R., Weber J.S. (2013). Exposure-response relationships of the efficacy and safety of ipilimumab in patients with advanced melanoma. Clin. Cancer Res..

[4] Borghaei H., Paz-Ares L., Horn L., Spigel D.R., Steins M., Ready N.E., Chow L.Q., Vokes E.E., Felip E., Holgado E. (2015). Nivolumab versus docetaxel in advanced non-squamous non-small cell lung cancer HHS public access. N. Engl. J. Med..

[5] Motzer R.J., Escudier B., McDermott D.F., George S., Hammers H.J., Srinivas S., Tykodi S.S., Sosman J.A., Procopio G., Plimack E.R. (2015). Nivolumab versus everolimus in advanced renal-cell carcinoma. N. Engl. J. Med..

[6] Chen D.S., Mellman I. (2017). Elements of cancer immunity and the cancer-immune set point. Nature.

[7] Lee Ventola C. (2017). Cancer immunotherapy, Part 1: Current strategies and agents. Pharm. Ther..

[8] Klener P., Otahal P., Lateckova L., Klener P. (2015). Immunotherapy approaches in cancer treatment. Curr. Pharm. Biotechnol..

[9] Rohaan M.W., Wilgenhof S., Haanen J.B.A.G. (2019). Adoptive cellular therapies: The current landscape. Virchows Archiv.

[10] Rosenberg S.A., Restifo N.P., Yang J.C., Morgan R.A., Dudley M.E. (2008). Adoptive cell transfer: A clinical path to effective cancer immunotherapy. Nat. Rev. Cancer.

[11] Al-Khami A.A., Mehrotra S., Nishimura M.I. (2011). Adoptive immunotherapy of cancer: Gene transfer of t cell specifcity. Self/Nonself Immune Recognit. Signal..

[12] June C.H. (2007). Principles of adoptive T cell cancer therapy. J. Clin. Investig..

[13] Rosenberg S.A., Dudley M.E. (2009). Adoptive cell therapy for the treatment of patients with metastatic melanoma. Curr. Opin. Immunol..

[14] Nguyen L.T., Saibil S.D., Sotov V., Le M.X., Khoja L., Ghazarian D., Bonilla L., Majeed H., Hogg D., Joshua A.M. (2019). Phase II clinical trial of adoptive cell therapy for patients with metastatic melanoma with autologous tumor-infiltrating lymphocytes and low-dose interleukin-2. Cancer Immunol. Immunother..

[15] Hong J.J., Rosenberg S.A., Dudley M.E., Yang J.C., White D.E., Butman J.A., Sherry R.M. (2010). Successful treatment of melanoma brain metastases with adoptive cell therapy. Clin. Cancer Res..

[16] Wang J., Shen F., Yao Y., Wang L.L., Zhu Y., Hu J. (2020). Adoptive cell therapy: A novel and potential immunotherapy for glioblastoma. Front. Oncol..

[17] Schmidt-Wolf I.G.H., Finke S., Trojaneck B., Denkena A., Lefterova P., Schwella N., Heuft H.-G., Prange G., Korte M., Takeya M. (1999). Phase I clinical study applying autologous immunological effector cells transfected with the interleukin-2 gene in patients with metastatic renal cancer, colorectal cancer and lymphoma. Br. J. Cancer.

[18] Hontscha C., Borck Y., Zhou H., Messmer D., Schmidt-Wolf I.G.H. (2011). Clinical trials on CIK cells: First report of the international registry on cik cells (IRCC). J. Cancer Res. Clin. Oncol..

[19] Garofano F., Gonzalez-Carmona M.A., Skowasch D., Schmidt-Wolf R., Abramian A., Hauser S., Strassburg C.P., Schmidt-Wolf I.G.H. (2019). Clinical trials with combination of cytokine-induced killer cells and dendritic cells for cancer therapy. Int. J. Mol. Sci..

[20] Zhang Y., Schmidt-Wolf I.G.H. (2020). Ten-year update of the international registry on cytokine-induced killer cells in cancer immunotherapy. J. Cell. Physiol..

[21] Giraudo L., Gammaitoni L., Cangemi M., Rotolo R., Aglietta M., Sangiolo D. (2015). Cytokine-induced killer cells as immunotherapy for solid tumors: Current evidence and perspectives. Immunotherapy.

[22] Chen D., Sha H., Hu T., Dong S., Zhang J., Liu S., Cao H., Ma R., Wu Y., Jing C. (2018). Cytokine-induced killer cells as a feasible adoptive immunotherapy for the treatment of lung cancer article. Cell Death Dis..

[23] Zhou Y., Chen C., Jiang S., Feng Y., Yuan L., Chen P., Zhang L., Huang S., Li J., Xia J.-C. (2019). Retrospective analysis of the efficacy of adjuvant CIK cell therapy in epithelial ovarian cancer patients who received postoperative chemotherapy. OncoImmunology.

[24] Zhang L., Xu Z., Chen X., Duan Y., Chen Z., Sun J. (2019). Clinical benefits of livin peptide-loaded DCs/CIKs combined with chemotherapy in advanced non-small cell lung cancer. Am. J. Cancer Res..

[25] Chen C.L., Pan Q.Z., Weng D.S., Xie C.M., Zhao J.J., Chen M.S., Peng R.Q., Li D.D., Wang Y., Tang Y. (2018). Safety and activity of PD-1 blockade-activated DC-CIK cells in patients with advanced solid tumors. OncoImmunology.

[26] Merhavi-Shoham E., Itzhaki O., Markel G., Schachter J., Besser M.J. (2017). Adoptive cell therapy for metastatic melanoma. Cancer J..

[27] Beavis P.A., Slaney C.Y., Kershaw M.H., Gyorki D., Neeson P.J., Darcy P.K. (2016). Reprogramming the tumor microenvironment to enhance adoptive cellular therapy. Semin. Immunol..

[28] Sackstein R., Schatton T., Barthel S.R. (2017). T-lymphocyte homing: An underappreciated yet critical hurdle for successful cancer immunotherapy. Lab. Investig..

[29] Deng X., Gao F., Li N., Li Q., Zhou Y., Yang T., Cai Z., Du P., Chen F., Cai J. (2019). Antitumor activity of NKG2D CAR-T cells against human colorectal cancer cells in vitro and in vivo. Am. J. Cancer Res..

[30] Gammaitoni L., Giraudo L., Leuci V., Todorovic M., Mesiano G., Picciotto F., Pisacane A., Zaccagna A., Volpe M.G., Gallo S. (2013). Effective activity of cytokine-induced killer cells against autologous metastatic melanoma including cells with stemness features. Clin. Cancer Res..

[31] Di Modugno F., Colosi C., Trono P., Antonacci G., Ruocco G., Nisticò P. (2019). 3D models in the new era of immune oncology: Focus on T cells, CAF and ECM. J. Exp. Clin. Cancer Res..

[32] Hirt C., Papadimitropoulos A., Mele V., Muraro M.G., Mengus C., Iezzi G., Terracciano L., Martin I., Spagnoli G.C. (2014). “In vitro” 3D models of tumor-immune system interaction. Adv. Drug Deliv. Rev..

[33] Weiswald L.-B., Bellet D., Dangles-Marie V. (2015). Spherical cancer models in tumor biology. Neoplasia.

[34] Nunes A.S., Barros A.S., Costa E.C., Moreira A.F., Correia I.J. (2019). 3D tumor spheroids as in vitro models to mimic in vivo human solid tumors resistance to therapeutic drugs. Biotechnol. Bioeng..

[35] Drost J., Clevers H. (2018). Organoids in cancer research. Nat. Rev. Cancer.

[36] Sherman H., Gitschier H.J., Rossi A.E. (2018). A novel three-dimensional immune oncology model for high-throughput testing of tumoricidal activity. Front. Immunol..

[37] Boucherit N., Gorvel L., Olive D. (2020). 3D tumor models and their use for the testing of immunotherapies. Front. Immunol..

[38] Brancato V., Oliveira J.M., Correlo V.M., Reis R.L., Kundu S.C. (2020). Could 3D models of cancer enhance drug screening?. Biomaterials.

[39] Menon J.U. (2018). 3D Tumor Models for Cancer Drug Discovery: Current Status and Outlook. J. Med. Therap..

[40] Kim I.S., Zhang H.-F. (2016). One microenvironment does not fit all: Heterogeneity beyond cancer cells. Cancer Metastasis Rev..

[41] Wang M., Zhao J., Zhang L., Lian Y., Wu Y., Gong Z., Zhang S., Zhou J., Cao K., Li X. (2017). Role of tumor microenvironment in tumorigenesis. J. Cancer.

[42] Jamal-Hanjani M., Quezada S.A., Larkin J., Swanton C. (2015). Translational implications of tumor heterogeneity. Clin. Cancer Res..

[43] Childs R.W., Carlsten M. (2015). Therapeutic approaches to enhance natural killer cell cytotoxicity against cancer: The force awakens. Nat. Rev. Drug Discov..

[44] Klingemann H., Boissel L., Toneguzzo F. (2016). Natural killer cells for immunotherapy—Advantages of the NK-92 cell line over blood NK cells. Front. Immunol..

[45] Fang F., Xiao W., Tian Z. (2017). NK cell-based immunotherapy for cancer. Semin. Immunol..

[46] Introna M. (2017). CIK as therapeutic agents against tumors. J. Autoimmun..

[47] Mesiano G., Grignani G., Fiorino E., Leuci V., Rotolo R., D’Ambrosio L., Salfi C., Gammaitoni L., Giraudo L., Pisacane A. (2018). Cytokine induced killer cells are effective against sarcoma cancer stem cells spared by chemotherapy and target therapy. OncoImmunology.

[48] Tonn T., Schwabe D., Klingemann H.G., Becker S., Esser R., Koehl U., Suttorp M., Seifried E., Ottmann O.G., Bug G. (2013). Treatment of patients with advanced cancer with the natural killer cell line NK-92. Cytotherapy.

[49] Sakamoto N., Ishikawa T., Kokura S., Okayama T., Oka K., Ideno M., Sakai F., Kato A., Tanabe M., Enoki T. (2015). Phase I clinical trial of autologous NK cell therapy using novel expansion method in patients with advanced digestive cancer. J. Transl. Med..

[50] Leuci V., Donini C., Grignani G., Rotolo R., Mesiano G., Fiorino E., Gammaitoni L., D’Ambrosio L., Merlini A., Landoni E. (2020). CSPG4-specific CAR.CIK lymphocytes as a novel therapy for the treatment of multiple soft tissue sarcoma histotypes. Clin. Cancer Res..

[51] Verneris M.R., Karimi M., Karami M., Baker J., Jayaswal A., Negrin R.S. (2004). Role of NKG2D signaling in the cytotoxicity of activated and expanded CD8+ T cells. Blood.

[52] Verneris M.R., Baker J., Edinger M., Negrin R.S. (2002). Studies of ex vivo activated and expanded CD8+ NK-T cells in humans and mice. J. Clin. Immunol..

[53] Seliger B., Ferrone S. (2020). HLA class I antigen processing machinery defects in cancer cells—Frequency, functional significance, and clinical relevance with special emphasis on their role in T cell-based immunotherapy of malignant disease. Methods in Molecular Biology.

[54] Dhar P., Wu J.D. (2018). NKG2D and Its Ligands in Cancer. Curr. Opin. Immunol..

[55] Introna M., Correnti F. (2018). Innovative clinical perspectives for cik cells in cancer patients. Int. J. Mol. Sci..

[56] Foty R. (2011). A simple hanging drop cell culture protocol for generation of 3D spheroids. J. Vis. Exp..

[57] Filippi-Chiela E.C., Oliveira M.M., Jurkovski B., Callegari-Jacques S.M., Silva V.D. da, Lenz G. (2012). Nuclear morphometric analysis (NMA): Screening of senescence, apoptosis and nuclear irregularities. PLoS ONE.

[58] Gammaitoni L., Giraudo L., Macagno M., Leuci V., Mesiano G., Rotolo R., Sassi F., Sanlorenzo M., Zaccagna A., Pisacane A. (2017). Cytokine-induced killer cells kill chemo-surviving melanoma cancer stem cells. Clin. Cancer Res..

[59] Berg S., Kutra D., Kroeger T., Straehle C.N., Kausler B.X., Haubold C., Schiegg M., Ales J., Beier T., Rudy M. (2019). ilastik: Interactive machine learning for (bio)image analysis. Nat. Meth..

